# Influence of intensified adherence counselling on viral load suppression of people receiving antiretroviral therapy at a health centre IV in southwestern Uganda: a qualitative study

**DOI:** 10.1186/s12981-021-00372-w

**Published:** 2021-07-28

**Authors:** Atwau Pius, Najjuma Nambi Josephine, Sibo Erick, Agoa Winifred, Mukashyaka Rita, Okoli Silverjoseph, Ezatiru Lucy, Nyemara Novatus

**Affiliations:** Faculty of Medicine, Mbarara University of Science And Technology, P.O. Box 1410, Mbarara, Uganda

**Keywords:** ART, Uganda, Adherence perceptions, Attitudes, Practices, Intensified Adherence Counseling (IAC)

## Abstract

**Background:**

The prevalence of Human Immunodeficiency Virus (HIV) among adults and children in Uganda is 6.2% and 0.5% respectively. The prevalence of viral load suppression in Uganda among all adults and children living with HIV is 59.6% and 39.3% respectively. High viral load compromises on the quality of life of an individual, and as well increases on the risk of transmission of the virus to the unborn for pregnant mothers and to the spouse. The UNAID 90-90-90 campaign recommends that 90% know their status, 90% get treatment and 90% have viral suppression. Non-adherence to Antiretroviral Therapy (ART) is one of the causes of the high viral load. The world health organization recommends Intensified Adherence Counselling (IAC) be given to all individuals with a high viral load. The perceptions, attitudes and practices of people receiving IAC is important in understanding how best IAC should be implemented.

**Methods:**

A qualitative study was done among 30 purposively selected individuals/guardians of children receiving ART and IAC at Kyabugimbi Health Center Four [HC IV (mini-hospital headed by a medical doctor)]. Data collected from Focus Group Discussion as audio-recordings in local language (Runyankole) was transcribed and later translated into English. Translated transcripts were analyzed manually using thematic content analysis.

**Results:**

The major themes identified include: adherence to ART; change of attitude towards ART after IAC, IAC expectation and outcomes, IAC and stigma, and improving social support through IAC for PLWHIV.

**Conclusion:**

Participating in IAC to be informative and led to change in their perceptions of HIV and better understanding the reasons for adherence to ART. IAC influenced the change in attitude and behavior thus seeking social support and mitigate stigma, which lead to a better ART adherence. However, there is need to improve on the way it is delivered both in structural setting and break down and packaging of information.

## Introduction

Adherence to life time treatment is challenging since one has to train themselves to consistently take medications for the rest of their life once they are diagnosed with HIV. Adherence becomes more complicated when an individual is expected to take medication even without obvious symptoms. Antiretroviral Therapy (ART) is important to achieve viral suppression and high CD4 cell counts, since HIV viral load suppression is the most important indicator of successful ART. It has been reported that ART side effects, non-disclosure, lack of improvement while on treatment, lack of knowledge on HIV, lack of assistance and forgetfulness, contribute to non-adherence [1–3]. ART adherence is the only way viral Load suppression can be achieved [2], thus improving quality of life. Despite the availability of ART, it has been reported that adherence to ART is a significant challenge in sub-Saharan Africa (SSA) [4–7]. The prevalence of viral load suppression in Uganda among all adults (15–64 years) and children (0–14 years) living with HIV is 59.6% and 39.3% respectively [8].

ART keeps the viral load low not to over whelm the immune system and this promotes healthy living for those living with HIV. Low viral load reduces chances of transmission [9]. If there is no adherence to ART there will be rise in viral load above 1000 copies/ml. Non adherence is indicated by a persistence high viral load. When a patient is initiated on ART they are monitored and supported to achieve better ART adherence. Intensified Adherence Counselling (IAC) also known as the Enhanced Adherence counselling has been recommended to improve viral suppression [10, 11]. World Health Organisation (WHO) and the Uganda ministry of HIV/AIDS guidelines have integrated IAC services at lower health facility where ART clinics are implemented [12, 13].

At Kyabugimbi health centre four [HC IV-(mini-hospital headed by a medical doctor)], all patients enrolled in ART clinic receive basic counselling on every visit for refill; however, for those with a high viral load receive the recommended IAC. It has been reported that a reasonable percentage of the patients report reduction in viral load after IAC while others reported a persistently high viral load even after IAC [14]. It is not known what individual and facility factors influence IAC. Therefore, this study was done to find out the perceptions, attitudes and practises of people with high viral load who were receiving IAC at a HC IV in southwestern Uganda.

## Methods

### Study design and setting

This is qualitative research study that was carried out between July and August 2020. Focus Group Discussions (FGD) were conducted to explore experiences of people living with HIV/AIDs (PLWHA) with high viral load receiving Intensified Adherence Counselling (IAC) at Kyabugimbi health center IV, Bushenyi district southwestern Uganda. FGD were used to allow the research solicit data from both participants shared experiences and differences in the group experiences. The local economy is mostly based on subsistence agriculture. Most of the people are ethnically Banyankole and the commonest language spoken is Runyankole. The FGD were conducted in Runyankole. The study was carried out at the ART clinic of Kyabugimbi Igara East sub district health center four (HC IV) in Bushenyi district. This health facility is 57.1 km west of Mbarara city. The clinic has a total of 780 individuals enrolled in the ART clinic. The clinic operates twice a week on Wednesday and Friday. Kyabugimbi HC IV is one of the two health centers IV in the district. It is a public health center and serves four sub counties surrounding it. The study focused on those with high viral load. On average there are about 60–70 people with a high viral load at each point in time.

The ART clinic is located in one of the rooms at the general out patient building. Patients have a large open waiting area that is shared with mothers attending the antenatal clinic. The counselling sessions for children takes place as a group in the waiting area; adults are counselled individually inside the ART room. The ART clinic is manned by 1(one) medical officer who is in charge of the clinic, 3(three) peer educators, and 1(one) counsellor who doubles as the ART clinic nurse and the facility data clerk for management of records.

### Study participants

This study intended to collect data from only those with a high viral load who were on IAC. Study was carried out among clients of the ART clinic of Kyabugimbi HC IV; these included all age groups who had a high viral load and had at least two test results.

### Sampling and data collection

Purposive sampling was used to select a homogenous group to participate in the FGD. In addition, are the two viral load measurements, we considered patients of the same sex, age, number of years on ART. Records were reviewed to identify those who had two test results with a high viral load tested within the recommended time. Participants were identified through the ART counselling nurse, PA and SE contacted these individuals over phone to request them to participate in the study.

We used a FGD guide based on literature about experience in adherence to aid data collection. It included questions about factors that affect adherence and influence of counseling, understanding of the ART, experience with ART and counseling, effect of counselling on adherence, impact on counseling and viral load suppression [1–3, 10, 11]. The guide was developed in English by the research team and was translated to Runyankole by a professional translator. A different professional translator translated the guide back to English to check for meaning, accuracy and conceptual equivalence. A final Runyankole guide was used to conduct the FGD.

A FGD guide was used. Each FGD had five to 10 people per group. FGD were facilitated by a professional qualitative research assistant who was neither known to the study participants nor part of the research team. During the FGD the facilitator was assisted by a note taker (assistant). The facilitator and note taker were fluent in Runyankole (local language). FGD participants included adults who had experienced IAC and care givers/guardians of children who had experienced IAC. FGD were conducted in Runyankole (local language), the discussions were audio recorded using a digital voice recorder. On average each FGD took 40 min. FGD were conducted until information redundancy occurred. Data collection and onsite analysis were done simultaneously to know when the saturation point was reached.

### Data management and analysis

The FGD audio-recordings were stored on flash disk after every FGD and later transferred to a password protected laptop. The audio recordings were then transcribed verbatim to text in Runyankole (Local Language) by the FGD facilitator and then translated to English by a professional translator. All the identifiers from the interviews were removed and replaced with the descriptions. Audio recordings were only accessed by the research team.

Thematic content analysis was used to analyze the data. Data were analyzed manually; two members (JNN and PA) read the transcripts to familiarize and each identified statements that pointed to address the research questions. Each member independently generated codes from the transcripts and all authors reviewed the codes. We used two stage of coding: initial and focused coding as described elsewhere [15, 16]. At the initial coding we broadly explored the experiences of participants with AIC and reviewed common features in the data. At focused coding we used inductive approaches to relate the codes generated from the initial coding. These formed the codes. The team agreed on the codes and merged related codes to form themes. Data is presented in sections under different themes with verbatim quotations.

### Human subject issues

The study proposal was reviewed and approved by Mbarara University Research Ethics Committee. The study was also registered by Uganda National Council of Science and Technology. We obtained individual written informed consent from all study participants. Consent forms were written in simple Runyankole (Local Language). Participants who were not able to read and write, used a thumbprint to confirm that they have understood the informed consent, and this process was witnessed by an individual who was able to read and write. We carried out the FGD in a private room at the hospital with less interruption and no intrusions. We assured that data collected was kept confidential by providing unique identifiers to identify the data. Only these participant identifiers were used on the audio files and transcripts. All research data was stored on a password protected computer and audio files were discarded after use. The FGDS were carried out by an independent trained qualitative research assistant who was not known to all participants. The research assistant was also fluent in the local language.

## Results

A total of 5 FGDs were conducted at Kyabugimbi health center four. A total of 30 respondents participated in the FGD. Their demographic characteristics are shown in the Table 1.

**Table 1:** Participants socio-demographics

	N%
Sex	
Male	14 (46%)
Female	16(53%)
Level of education	
No formal education	3 (10%)
Primary school level	21(70%)
Secondary school level	6 (20%)
Marital status	
Never married	6 (20%)
Married or cohabiting	8 (26%)
Divorced or separated	4 (13%)
Widowed	3 (10%)
Not applicable	9 (30%)
Viral load result	
Less than 50 copies/ml	10 (33%)
Between 50 and 1000 copies/ml (controlled)	4(13%)
Above 1000 copies/ml	16 (53%)

Children were represented by their care givers (care giver was the person responsible for monitoring child’s treatment, collecting child’s medications). The themes and sub themes are summarised in the Table 2.

**Table 2:** Summary of qualitative results from

Theme	Sub themes
Change of attitude towards ART after IAC	Understanding the importance of adherence after IAC
	Attending review/refill appointments
	Overcoming fears and myths related to ART
IAC expectations and outcomes	Improved adherence on ART
	Emphasis on timing for ART
	Client counselor relationship
	Time for taking the ART
IAC and stigma	School affecting ART
	Habit of taking ART
	Separation and isolation of ART clinic for privacy
Improving social support through IAC for PLWHIV	Care giver involvement
	Mode of counselling; individual versus group
	Habit of taking ART
	HIV status disclosure

### Change of attitude towards ART after IAC

Participants explained that their attitudes towards ART changed after they had received IAC. Before receiving IAC, participants explained that their attitudes towards ART was difficult without understanding what ART was for. It was reported that taking ART is filled with a lot of fears and worries, many misconceptions and a feeling of life coming to an end. Patients were being filled with difficulties of thoughts and decision making, and this greatly impacted their adherence to ART and thus their quality of life.

“One would think that even if they swallow this medicine, one will reach in the middle and get fed up and give up on taking the medicine and wait for death. But later, when we started taking the medicine and after the nurse talking to us, we later realized that what we thought is not what actually happens”. (FGD-2, Male, 7 years on ART)

IAC was reported to be very beneficial to the care takers and guardians of children on ART. They reported that through IAC they received more knowledge:

“Sometimes I would not even give it to the child because the medicine appeared to be too much…But when I kept coming here during the training/seminars, I also started learning the cause of the problem.” (FGD 2, participant 3)

Lack of adequate knowledge about ART contributed to non-adherence. Understanding the importance of continuing to take ART and its benefits to one’s health was reported as a motivator to adherence. Patients claimed that when it seemed like the health worker was the only one who had knowledge about the ART, they were hesitant to take it.

### IAC expectations and outcomes

IAC is one of the interventions that are given to PLWH with poor viral suppression. It was reported that IAC improved adherence to ART. Factors that affect ART adherence were found to be both patient centered and health care system generated. Some of the patient centered reasons that lead to non-adherence is the fear of side effects of ART, perceived harm of ART, and pill burden for patients that had other comorbidities. On the other hand, factors from the health care system included medications being out of stock and group counselling.

Upon receiving IAC, study participants reported that they loved their medications because they were seeing great improvement when they took their drugs correctly. This is because they were taught how to take them through the counselling sessions that they received during IAC. This better adherence to the ART resulted to lower viral load, thus better health. One of the FGD revealed that:

“I was given enough counselling and medicine which I started taking immediately. Nowadays, I no longer fall sick all the time like I used to before I started taking medicine correctly. I always take my medicine as prescribed and now I can feel an improvement”. (FGD 2; 17- year-old male, 9 years on ART)

“The reason why I accepted that the ART drugs really work was this child was really very weak with loose stools body itching all the time. I used local herbs tried to feed it but no improvement really but when it started its art medications and I also learnt how to give it the medications, the child is very well and I knew that that these drugs are very useful” (FGD 4; 41-year-old female, 11 years on ART)

One of the participants stated that:

“so, coming to the hospital and they advise us on ARVs is so informative because as a person I was taking the medications wrongly” (FGD 1; 43 years old female, 7 years on ART)

Another participant said that, IAC sessions helped her have their viral load monitored and worked to reduce it;

“But after giving me counselling, I understand why I need to take my medications and I see now my viral load is reducing” (FGD 1, 45-year-old female, 4 years on ART*)*

Most of the patients reported that there was failure for viral load suppression after initiation of ART. This was because of poor adherence that was caused by patient centered reasons. Through IAC they come to realize the importance of prescription instructions through the emphasis made by their nurse.

Patients expressed a need for a comprehensive support in their care, they needed a day-to-day guidance on how to cope with the new life, they felt the necessity for them being taught all that is necessary to help them live well with the new condition.

“For example, if I was required to take the medicine at 7:00pm. I would not take it until after prep, at around 10:00pm. If it so happened that after prep, the students around me are so many, I would wait and swallow the medicine at around 11:00pm when everybody else has slept. As a result, I faced a challenge and started suppressing on time until to date because I was not taking the medicine at similar times”. (FDG 2, male, 9 years on ART)

### Improving social support through IAC for PLWHIV

It was reported that participants picked their medications from the ART clinic whenever available. It was also reported that the health care workers did not have a system in place to monitor or ensure that the ART picked was being taken by the patient, except for viral load monitoring and what the patient reported. In situations where participants were not able to remember when to take their medications health care providers identified a social support system. IAC helped patients realize the importance of HIV status disclosure. Disclosure was emphasized to help the participants get support from the people they trust for better adherence.

Before IAC they were not willing to disclose their status, but after it was proposed to them during the IAC sessions and its benefits discussed, participants shared their HIV status with one or two of their close relatives. One of the male participants emphasized that:

“She asked me about the people I stay with at home, I told her and she asked me that can you really tell people that you stay with at home about your status, I said yes. So, I went home and told them and the response was good and they help me with taking my medications” (FGD 4 38-year-old male, 4 years on ART).

### IAC to stop stigma towards PLWHIV

Stigma still continues being an issue affecting adherence hence high viral loads. Participants mentioned that there’s always a will to adhere to the medications, although this is sometimes affected by stigma. Even when the patients knew the benefits of ART to some reasonable extent but do not like to be pointed at as HIV positive. Participants reported that living with HIV feels like a death sentence, being sick is not a normal thing to a human and its clear to everyone that anyone taking any kind of medication must be sick; so, people get concerned and want to know what one could be suffering from. With this assurance of people around at one point getting concerned, those living with HIV tend to avoid that (ART medications) which attracts such questions in order to freely socialize with other people;

“When I started secondary school, the school I went to had no one to follow me up. I used to keep those tablets in my suit case and I would never swallow these tablets in the presence of others”. (FGD 2, male, 9 years on ART)

Getting it known to the people around those living with HIV is an important source of support to help them take their medications well. It is however not a common practice by PLWHIV to let their significant others know about their status and therefore it is difficult for them to follow their time for taking the medications since they keep doing it in hiding and this causes suboptimal adherence which results in high viral load.

“Okay there are things that come in and disrupt us when we are taking these medications. Sometimes you have a husband and you are scared of telling him about your status to him so it makes you to medication wrongly and it really leads to an increase in the viral load”. (FGD 1, female, 4 years on ART)

## Discussion

In this study, most of the participants have high viral load not due to virologic failure. ART non adherence is one of the major causes of high viral load [17]. Adherence to ART is required for best outcomes, although this is not always achieved for different reasons. We found out that most the reasons and challenges that lead to non-adherence were addressed through IAC. Sometimes patient did not know, the problem that contributed to their non-adherence. Patients associated their high viral load to factors that they got to know after they had had session of intensified adherence counselling for example the way they took their medications, some had difficulties in following a fixed time to take their medications, others had an interference on their routine of taking medications whenever they had challenges concerning them.

The results of the study indicate that participants were more ready to share their status, get more social supports and also reduce stigma. Stigma has been reported to affect adherence, in a study carried out in India, It was found out that internalized stigma affected adherence among rural women [18]. This is consistent with our finding were stigma hindered participants adherence and interventions to solve this stigma would improve adherence.

The study results indicated that those living with their families or relatives who were aware of their HIV status received support. This support included reminding them when they are supposed to take their medications and counseling them about ART. Disclosure and having a supportive family member is known to improve adherence to ART [19]. This is also consistent with other earlier studies.

Therefore, there is need to get people involved in the care of their relatives who are positive; though it is challenging with the fact that HIV is a highly stigmatized disease so even the relatives do not wish to be identified with their relatives who are HIV positive. However, HIV positive people feel free relating with their colleagues who are also positive so it is important to strengthen support group structure for HIV positive people.

Perceptions and understanding of individuals about ART so much influences their behavior and attitudes to ART which attitudes impact on their adherence to taking their medications [20]. Patients did not have a good understanding of reasons why they needed treatment. Many had miss perceptions on ART thus were not willing to take ART medications due to the fear of being affected by what they perceived the drugs would cause to them.

### Study limitations

One of the limitations from this study is that is the finding are for a qualitative study there is need for quantification of the results to understand the true effect of IAC and conclude. Another limitation was that the study was done in a single setting in southwestern Uganda, the results may not reflect the experience of People living with HIC receiving AIC in other settings.

## Conclusion

In conclusion, participating in IAC was informative and led to change in their perceptions of HIV and better understanding the reasons for adherence to ART. Participants improved strategies practices of health changed and they developed a good attitude towards ART. Intensified adherence counselling has an impact on behavior of PLWHIV of which behavior influences adherence to ART. However, IAC cannot stand alone without a proper supportive structure for patients after receiving it. We recommend extension of IAC to peer support groups. Social support for people living with HIV is a continuation of the care they receive from health facilities and deals more with supporting them psychologically which is less addressed by the ART medications.

## Data Availability

Data and research materials are available on request from the corresponding author on reasonable request.

## Abbreviations

ART: Antiretroviral therapy
FGD: Focus group discussion
HC IV: Health center four
IAC: Intensified Adherence Counselling
MUST: Mbarara University of Science and Technology
PLWHIV: People living with HIV
UNCST: Uganda National Council for Science and Technology
WHO: World Health Organization
